# Real-world experience with pro re nata dosing of intravitreal dexamethasone implant for eyes with refractory diabetic macular edema

**DOI:** 10.3205/oc000148

**Published:** 2020-04-08

**Authors:** Pukhraj Rishi, Ekta Rishi, Yamini Attiku, Abhinav Dhami, Vandana Iyer

**Affiliations:** 1 Shri Bhagwan Mahavir Vitreoretinal Services, Sankara Nethralya, Chennai, India

**Keywords:** diabetes mellitus, macular edema, recalcitrant diabetic macular edema, intravitreal injection, dexamethasone, Ozurdex®

## Abstract

**Aims:** To evaluate treatment outcomes of pro re nata dosing of intravitreal dexamethasone implant in eyes with refractory diabetic macular edema (DME) amongst Indian subjects.

**Methods and material:** Retrospective, interventional case series. Medical records of 28 eyes of 23 patients with refractory DME who underwent intravitreal dexamethasone (700 µ) implant were reviewed.

Paired t-test was carried out to measure mean change in the parameters evaluated. Mann-Whitney U test and Fisher’s exact t-test were done to explore differences between groups receiving single or multiple injections.

**Results:** Best corrected visual acuity (BCVA) and central macular thickness (CMT) at baseline were 0.85 (±0.44) and 612 µm (±123), respectively. Mean CMT over 6 months (measured monthly) following injection was 340±119 µm (p=0.001), 346±150 µm (p=0.02), 368±169 µm (p=0.02), 304±174 µm (p=0.001), 525±216 µm (p=0.94) and 532±201 µm (p=0.46), respectively. Mean BCVA at each month following injection was 0.68±0.36 (p=0.02), 0.75±0.45 (p=0.42), 0.55±0.40 (p=0.11), 0.63±0.40 (p=0.12), 0.78±0.30 (p=0.90) and 0.60±0.47 (p=0.92), respectively. Mean follow-up was 12 months (range: 6–33 months). Mean BCVA and CMT at mean 12 months were 0.72±0.46 (p=0.10) and 358 µm±189 (p=0.0001), respectively. Seven eyes had raised IOP; five eyes required cataract extraction.

**Conclusions:** Intravitreal dexamethasone implant is effective in treatment of refractory DME. However, its therapeutic effect lasts for about 4 months.

## Introduction

Diabetic macular edema (DME) is the second most common cause of persistent severe visual loss in patients with diabetes [1]. Laser therapy, which was previously considered as the gold standard for the management of DME, has its own limitations as it may produce paracentral scotomas, impaired color vision, and decreased contrast sensitivity [2], [3]. Apart from these, a subset of patients does not respond to laser therapy, and the term ‘refractory’ macular edema has been employed for such cases. Nearly 26% of patients with DME in Early Treatment Diabetic Retinopathy Study (ETDRS) were found refractory to laser therapy and suffered from progressive loss of vision despite multiple laser sessions [1]. Of late, the focus of treatment for DME has included varied anti-vascular endothelial growth factor (VEGF) agents, with several studies showing their therapeutic superiority over laser monotherapy [4], [5], [6], [7]. Anti-VEGF therapy is now the treatment of choice for centre-involving DME. Even as experience with anti-VEGF agents increases, reports of ‘non-responders’ continue to emerge [8], [9]. In the recently completed RESTORE extension study which treated 240 eyes with ranibizumab pro re nata, 14% of the patients had persistent macular edema at the end of a three-year follow-up [10]. The underlying causes are attributed to the varied pathogenetic mechanisms responsible for causing macular edema in diabetes including secondary changes in tight junctions, loss of pericytes, endothelial cell loss, retinal vessel leukostasis, upregulation of vesicular transport and inflammatory cells, and increased permeability of surface membranes of retinal vessels and retinal pigment epithelium [11]. There are even reports of tachyphylaxis with ranibizumab (Genentech Inc, San Fransisco, CA, USA) and bevacizumab (Genentech Inc, San Fransisco, CA, USA) [12].

Recently, the focus on VEGF-dependent mechanisms has somewhat overshadowed VEGF-independent pathways possibly involved in the pathogenesis of refractory DME. Corticosteroids are believed to reduce macular edema through a more widespread action that blocks VEGFs, inflammatory cytokines, and prostaglandins and could play a pivotal role in the management of such refractory DME cases [13]. The dexamethasone drug delivery system implant (Ozurdex^®^, Allergan Inc, Irvine, California, USA) provides sustained levels of dexamethasone in the vitreous and has been evaluated in a few reports for the management of DME [14], [15], [16], [17], [18]. A Pubmed search using the keywords <refractory/recalcitrant diabetic macular edema><dexamethasone> and <Ozurdex> revealed nine relevant studies [15], [16], [18]. Moreover, the effects of pro re nata (PRN) dosing of Ozurdex^®^ in DME are yet to be established. In this study, we report a series of 28 eyes with refractory DME treated with PRN Ozurdex^®^ therapy and a mean follow-up of 12 months (range: 6–33 months).

## Patients and methods

This was a single centre, retrospective, interventional case series in a tertiary eye care centre. Prior approval by the institutional review board (IRB) was obtained. All patients were provided written informed consent forms and consented after a detailed explanation of the nature of the drug, and the risks and benefits of the treatment. The study adhered to the tenets of the Declaration of Helsinki. Case records of patients diagnosed with DME from January 2011 to December 2013 were retrieved from the electronic database. In this period, 239 eyes were diagnosed of DME. Of these, 42 eyes had refractory DME and were treated with intravitreal Ozurdex^®^ implant. Of these 42 eyes, 11 eyes were excluded as they could not complete the six-month follow-up. Of the remaining 31 eyes, three patients underwent retreatment with either intravitreal ranibizumab, bevacizumab or triamcinolone acetonide (Tricort, Cadilla Pharmaceuticals Ltd, Ahmedabad, Gujarat, India) and were excluded from the study. The remaining 28 eyes with a follow-up of at least 6 months (mean: 12 months, range: 6–33 months) were included in the study.

Inclusion criteria allowed patients with refractory diabetic macular edema who met the following criteria: age older than 18 years, persistent macular edema involving the center of the fovea for 3 or more months after at least three consecutive intravitreal anti-VEGF injections (Refractory DME), and a minimum follow-up of 6 months. A less than 10% decrease in CMT at 1-month follow-up was considered as lack of response to treatment. Exclusion criteria included a history of corticosteroid-responsive intraocular pressure (IOP) rise, any intraocular surgery up to 3 months before the initial Ozurdex^®^ injection and use of any other intravitreal agent apart from Ozurdex^®^ during the study period. Eyes with macular tractional component (epiretinal membrane or vitreomacular traction) were also excluded. Baseline characteristics of subjects included in the study are listed in Table 1 (Tab. 1).

**Figure 1:**
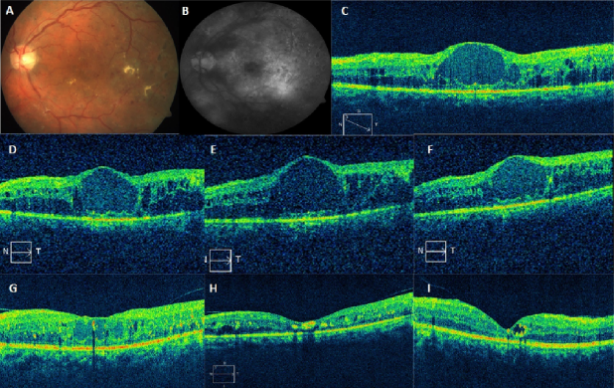
Representative case of a 56-year-old male with recalcitrant DME in the left eye (A) and old laser photocoagulation marks at baseline; visual acuity was 6/60. Diffuse DME is observed on late phase angiogram (B) and cystoid changes with macular thickening is seen on OCT (CMT=688 µm) (C). (D, E, F) Serial OCT images following monthly injection of intravitreal bevacizumab (1.25 mg/0.1 ml) with persistent edema (CMT=650 µm; 732 µm and 768 µm, respectively). OCT images (G, H, I) after 1, 2, and 5 months of intravitreal dexamethasone implant (CMT=328 µm; 200 µm and 125 µm, respectively). There is marked reduction in macular edema (G), the foveal contour is restored (H), the foveal contour is maintained with a persistent intraretinal cyst (I).

A comprehensive ophthalmic history was elicited from all the patients. Clinical examination included best-corrected logMAR visual acuity (BCVA), applanation tonometry, anterior segment examination including evaluation of lens status, dilated fundus examination, and optical coherence tomography (OCT) of the central macula showing macular thickness at baseline and subsequent follow-up visits. All eyes underwent intravitreal dexamethasone implant (Ozurdex^®^) injection under sterile precautions in the operating room. Patients were prescribed topical ciprofloxacin 0.3% (Cipla Ltd, Mumbai, India) six times/day for 3 days prior to and 5 days following injection. Patients were followed up every month for the first 6 months after intravitreal Ozurdex^®^ injection (Figure 1 (Fig. 1)). Thereafter, follow-up intervals were gradually extended at the discretion of the treating physician. Re-treatment was advised if the following criteria were met: a) CMT>250 µm on OCT, and/or b) visual acuity decline of 2 Snellen lines [18]. The need for adjunct laser therapy was left at the treating surgeon’s discretion (Table 2 (Tab. 2)). Primary outcome measures were change in best-corrected visual acuity (BCVA) and CMT from baseline at each month up to 12 months on average. Secondary outcome measures included change in IOP, progression of cataract, and occurrence of any other side effects due to the implant. Study eyes were classified into two groups based on whether a single injection (n=15, group A) or multiple injections (n=13, group B) were required.

Paired t-test was carried out to measure mean differences between pre- and post-injection values of the parameters evaluated (logMAR and CMT) and obtained at different follow-up visits. Mann-Whitney U test was done to explore the differences between the group of eyes that had a single (n=15, group A) injection versus the group of eyes that had multiple injections (n=13, group B). A p value of <0.05 was considered statistically significant. For comparing the differences in the systemic associations between the two groups, Fisher’s exact t-test was done (Table 3 (Tab. 3)).

## Results

Twenty-eight eyes of twenty-three patients were included in the study. There were 20 male and 3 female patients, all with type II diabetes mellitus. The average age was 56.5 years (median: 57 years, range: 26–79 years). Mean baseline HbA1c was 6.8%. The average duration of DME was 14 months (median: 7 months, range: 6–96 months). All eyes (n=28) included in the study had received prior therapy for DME and were refractory as they had not responded to laser/anti-VEGF therapy. Details of prior therapy received, diabetic retinopathy grading, associated systemic diseases, pre-treatment IOP values, and lens status are listed in Table 1 (Tab. 1).

In all, 45 intravitreal Ozurdex^®^ injections were given in the study period; twenty-eight as primary and seventeen as re-injections (mean: 12, range: 6–33 months). Thirteen eyes were re-injected. The mean number of injections required in group B was 2.3. Of the 17 re-injections, eight (27%) were given at a mean interval of 5 months (median: 4, range: 4–6 months), five (17%) were given after a mean interval of 9 months (median: 9, range: 8–18 months) and four (13%) were given after a mean interval of 18 months (median: 18, range: 18–28 months) following the primary injection.

**Figure 2:**
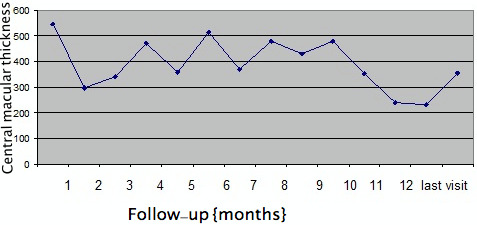
Monthly changes in central macular thickness (CMT) in microns after Ozurdex^®^ injection for refractory diabetic macular edema in 28 eyes

**Figure 3:**
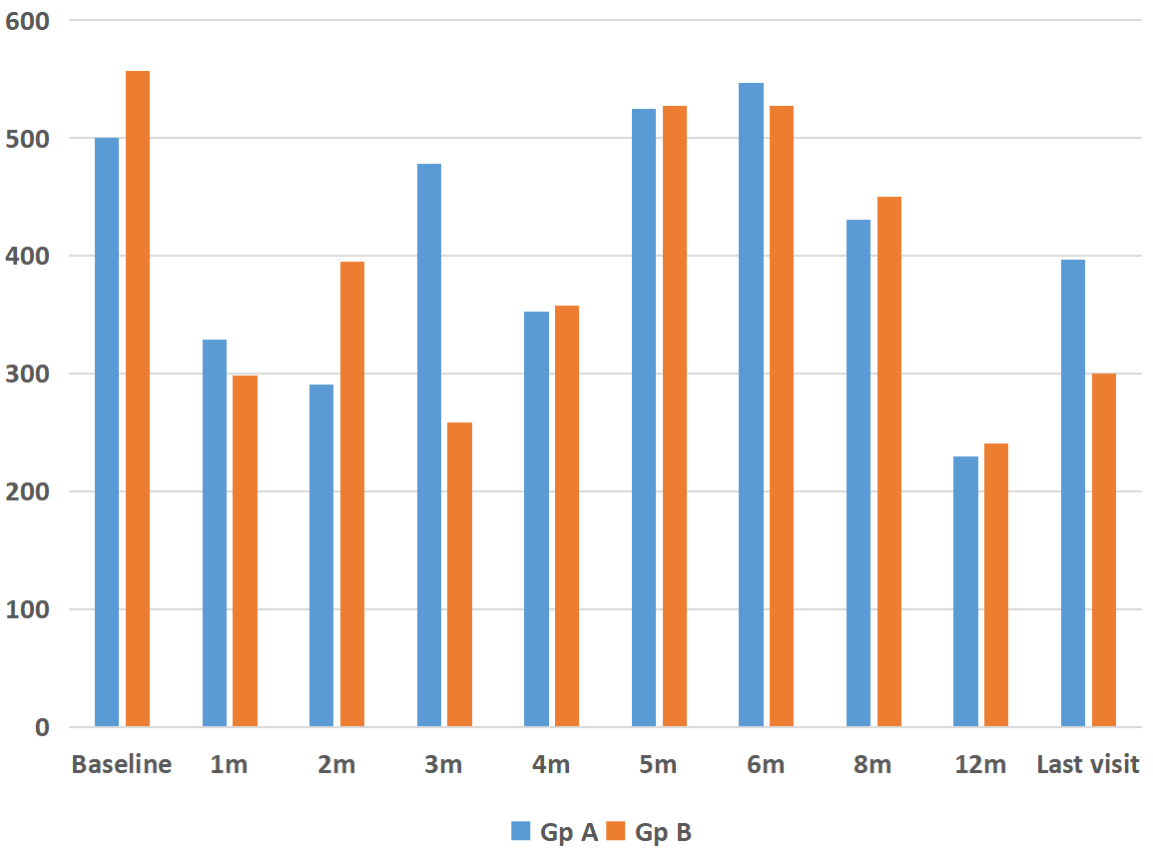
Comparison of changes in central macular thickness (CMT) after Ozurdex^®^ injection for refractory diabetic macular edema in eyes with single (group A) and multiple (group B) injections

The mean CMT at baseline was 612±123 µm, which reduced to 340±119 µm at month 1 (P=0.001). This was sustained at month 2 (346±150 µm, P=0.02), month 3 (368±169 µm, P=0.02) and month 4 (304±174 µm, P=0.001). There was a rebound increase in CMT at 5 months (525±216 µm, P=0.94) which remained the same at month 6 (532±201 µm, P=0.46), respectively (Figure 2 (Fig. 2)). However, on long-term (mean 12 months) follow-up, CMT was well controlled with PRN dosing schedule and was 358±189 µm (P=0.0001) (Figure 2 (Fig. 2), Figure 3 (Fig. 3)). Twenty-five eyes (group A=13, group B=12) had subfoveal fluid at presentation, of which 18 eyes showed resolution of SRF with treatment. Figure 1 (Fig. 1) demonstrates the effectiveness of Ozurdex^®^ implant in a case of recalcitrant DME. Time intervals for re-injection of implant are mentioned in the section on ‘additional treatments’.

**Figure 4:**
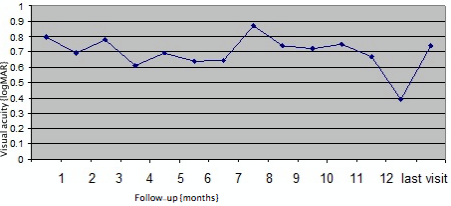
Monthly changes in logMAR visual acuity after Ozurdex^®^ injection for refractory diabetic macular edema in 28 eyes

**Figure 5:**
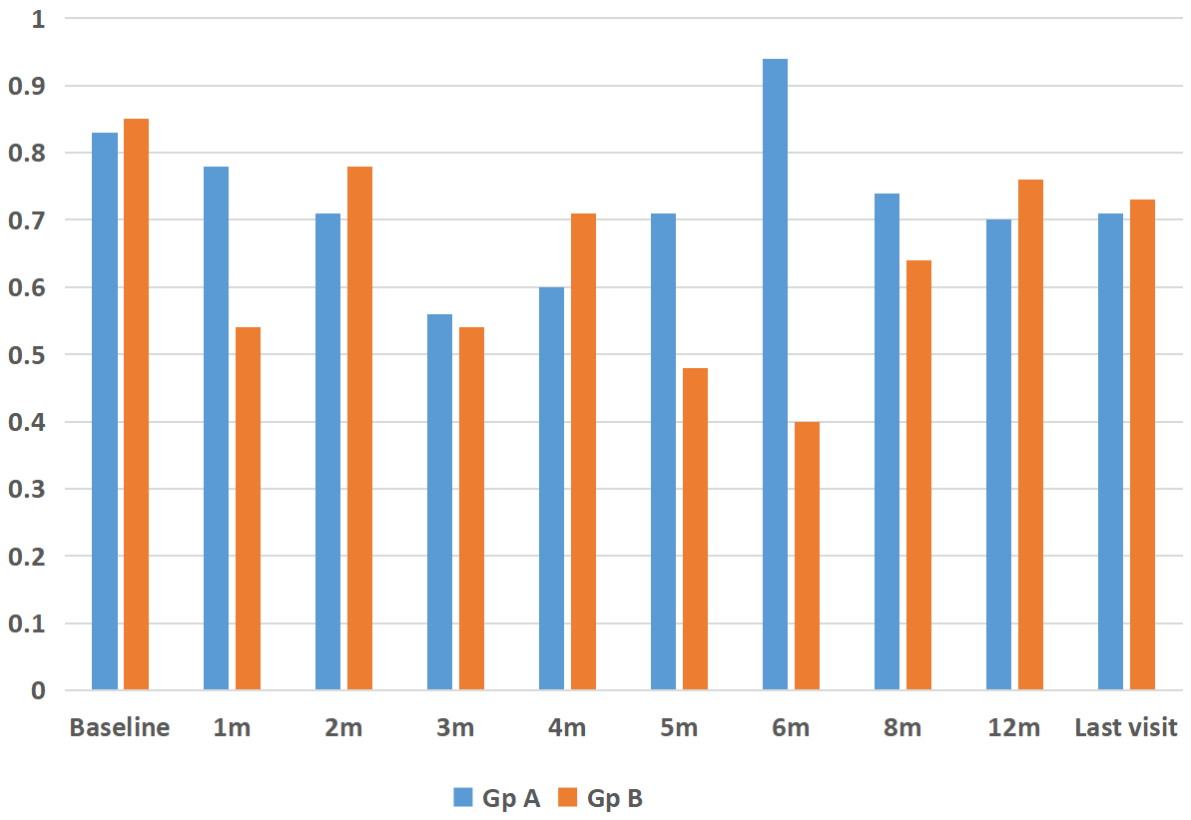
Comparison of changes in logMAR visual acuity after Ozurdex^®^ injection for refractory diabetic macular edema in phakic and pseudophakic eyes at baseline

BCVA at baseline was 0.85±0.44 logMAR. Overall, BCVA in the first six months was as follows (Figure 4 (Fig. 4)):

- month 1: 0.68±0.36 logMAR (P=0.02),
- month 2: 0.75±0.45 logMAR (P=0.42),
- month 3: 0.55±0.40 logMAR (P=0.11),
- month 4: 0.63±0.40 logMAR (P=0.12),
- month 5: 0.78±0.30 logMAR (P=0.90) and
- month 6: 0.60±0.47 logMAR (P=0.92),

respectively. After the initial six one-monthly interval visits, patients were followed up for a mean 12 months (median: 13 months, range: 6–33 months). Visual acuity at mean 12 months follow-up was 0.72±0.46 logMAR (P=0.10). There was no statistically significant difference between the group of eyes that had a single implant (n=15) versus the group of eyes that had multiple implants (n=13) in terms of visual outcomes (P=0.21, Figure 4 (Fig. 4), Figure 5 (Fig. 5)).

**Figure 6:**
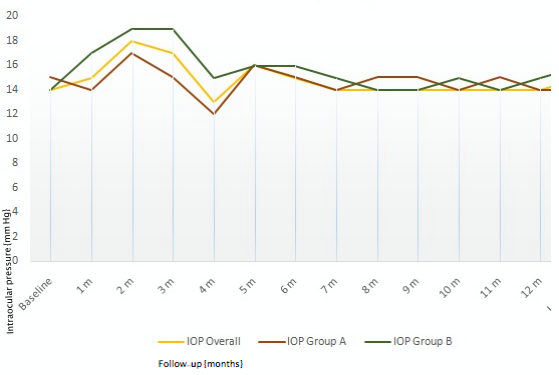
Mean monthly changes in IOP among study eyes

**Figure 7:**
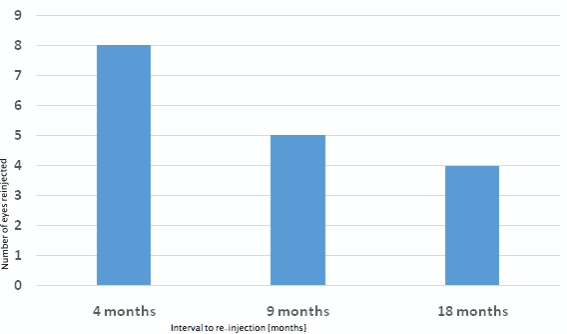
Mean interval to re-injection

Seven eyes had increased IOP following Ozurdex^®^ injection (mean=25 mmHg, range: 22–29 mmHg) (Figure 6 (Fig. 6)). Two of seven eyes had single injection while five of seven eyes had multiple injections. Mean interval to re-injection is shown in Figure 7 (Fig. 7). However, rise in IOP in all the five eyes treated with multiple injections was observed only following the first injection. All seven eyes were treated with topical anti-glaucoma medication (AGM) (mean 1 drug), and IOP was restored to normal. AGM was continued as long as the implant was visible in the vitreous cavity in patients who received single injection, while AGM was continued during the follow-ups in patients who received multiple injections.

Progression of cataract required surgery in 5 (18%) eyes (group A=2; group B=3). Among other notable adverse effects, epiretinal membrane developed in 6 (21%) eyes, while one (3%) eye developed vitreous hemorrhage two months after injection; both events may well have been related to the natural progression of the disease.

Cataract progression was observed in 5 (18%) eyes, four of which required surgical extraction that resulted in significant visual improvement (Table 2 (Tab. 2)). One (4%) eye required surgical extraction during the first 6 months, while 4 eyes (14%) underwent cataract extraction after a mean interval of 9 months (median: 9 months, range: 7–11 months) from the first injection. Progression was faster in eyes which received multiple implants. One eye underwent vitrectomy for vitreous hemorrhage (Table 2 (Tab. 2)).

Seventeen re-injections were performed during the study period at the discretion of the treating physician, chiefly based on CMT (Table 2 (Tab. 2)). In 8 eyes, Ozurdex^®^ was injected twice, in 1 eye it was injected thrice, while 4 eyes had 4 intravitreal injections of Ozurdex^®^. All five cases of PDR required a single injection (Table 3 (Tab. 3)). Adjunct macular laser photocoagulation was also done in 11 (39%) eyes: 2 eyes in group A, and 9 eyes in group B. One patient was treated at 1-month, 2 at 3-month, 2 at 4-month, 4 at 5-month and 2 at more than 6-month follow-up. At last visit, the mean logAR VA was 0.6 logMAR in patients who received adjunct laser, while it was 0.83 logMAR in patients who did not receive laser.

## Discussion

The role of low-grade inflammation in the pathogenesis of diabetic macular edema (DME) has drawn increasing attention [19]. Clinical observations report a subset of eyes that do not respond favorably to laser and/or anti-VEGF therapy. Hence, steroids have emerged as a treatment option in such eyes in the management of DME [20], [21], [22], [23]. Intravitreal triamcinolone has been shown to be effective but the inadvertent side effects, cataract progression, and rise in IOP limit its widespread use [20]. Our study reveals that the central macular thickness is seen to decrease from the first month after the injection and the drug effect is sustained for four months. Visual acuity showed statistically significant improvement from baseline to one-month after injection and shows a trend towards improvement up to mean 12 months. This slow improvement may be due to the fact that cataract was increasing, but 5 eyes had cataract surgery during the study period, somehow offsetting the adverse effect. It appears that the maximal therapeutic effect in terms of visual gain is derived in the initial month and sustained thereafter for four months in comparison to the baseline VA. Previous reports have shown similar results [24], [25]. Further visual improvement was limited and did not correlate with the continuous improvement in central macular thickness. This could be attributed to the chronic nature of macular edema (mean duration: 14 months, range: 3–96 months) resulting in limited functional recovery. About 40% reduction in CMT from baseline was observed one month after Ozurdex^®^ injection. This effect was sustained for a period of 14–16 weeks. Similar findings were reported in another study, albeit in vitrectomized eyes [24]. They reported the peak effectiveness of Ozurdex^®^ implants between 8 and 13 weeks after injection, wherein the mean CMT reduced by 27% to 39%. In our study, CMT reduced by 36% at the end of the 12-months follow-up period. Previous reports have demonstrated the efficacy of Ozurdex^®^ in reducing macular edema due to DME [14], [15], [16], [17], [18], [26], [27], [28]. The UDBASA study has shown the individualized PRN regimen to have better anatomical and functional outcomes than fixed regimens in DME at 6 months [29]. Our study demonstrates the efficacy of Ozurdex^®^ in treatment of refractory diabetic macular edema over a mean follow-up of 12 months.

Rise in intraocular pressure and progression of cataract are well-documented adverse effects of corticosteroid therapy. Reports with the use of intravitreal triamcinolone acetonide for the treatment of diabetic macular edema have shown that 44% of the patients required anti-glaucoma medications at 2-year follow-up [20]. In the Geneva study, 25% of the patients receiving intravitreal dexamethasone (0.7mg) required IOP lowering medication, and less than 16% of the eyes had an increase in IOP to ≥25 mmHg at day 60 [25]. The study found that this rise in IOP was rather a transient effect and there was no difference between the dexamethasone implant groups and the sham group by day 180. In our study, 25% of patients showed a rise in IOP and all were controlled effectively with IOP lowering drugs. An expert panel of European ophthalmologists concluded that the increased IOP following intravitreal steroids in DME was controlled in the majority of the cases by antiglaucoma medications and laser trabeculoplasty [29]. It was shown that patients with pre-existing glaucoma needed antiglaucoma medication following intravitreal Ozurdex^®^ for control of IOP [30]. Rate of cataract formation 12 months after Ozurdex^®^ injection had been previously reported to be as high as 29.8%, depending on the number and dosage of injections [25]. In our study, 18% of patients showed progression of cataract requiring surgery up to a mean follow-up of 12 months. No statistically significant difference between the two groups in terms of visual outcomes (p=0.21) could be reached, and this might be due to the small sample size. The other major limitation of this study is its retrospective nature. Nevertheless, our study provides an insight on the short-term efficacy and safety measures related to Ozurdex^®^ in eyes with refractory DME. We feel that poor visual gain in our series was due to the chronic nature of the disease (recalcitrant DME) and had nothing to do with response to Ozurdex^®^. But having said this, we recommend earlier inclusion of Ozurdex^®^ in the treatment algorithm, especially in pseudophakic eyes and in eyes with clumps of macular hard exudates at presentation. 

## Conclusions

In conclusion, Ozurdex^®^ was found effective in significant reduction of CMT in eyes with refractory diabetic macular edema. However, further large prospective studies are required to validate our results and determine the optimal retreatment interval with Ozurdex^®^ implant.

## Notes

### Competing interests

The authors declare that they have no competing interests.

### Acknowledgements

We acknowledge the contributions of Mr. Vishwanathan, Department of Biostatistics, Medical Research Foundation, Sankara Nethralaya, Chennai, India to the statistics in this manuscript.

## Figures and Tables

**Table 1:**
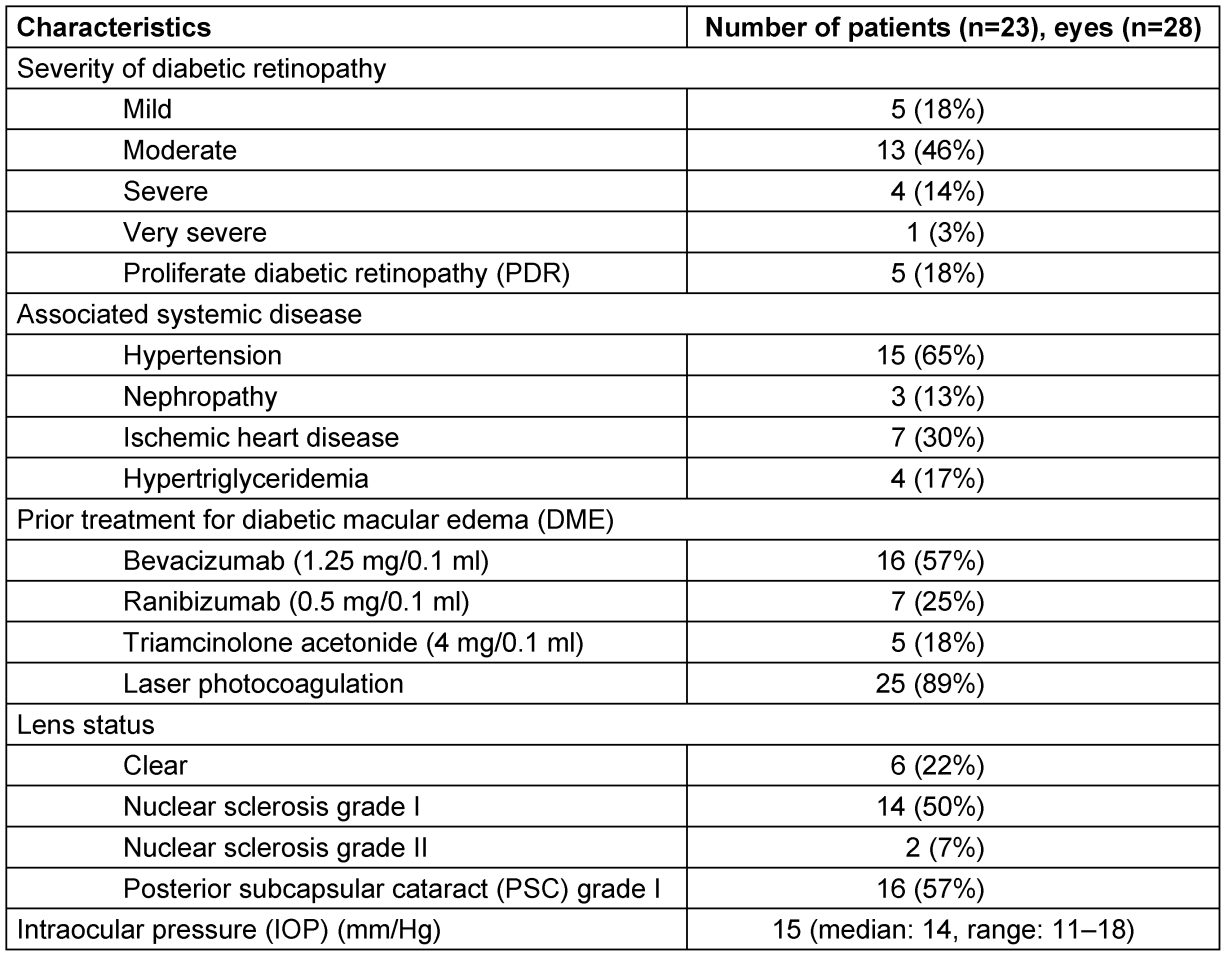
Intravitreal dexamethasone implant for refractory diabetic macular edema: baseline characteristics

**Table 2:**
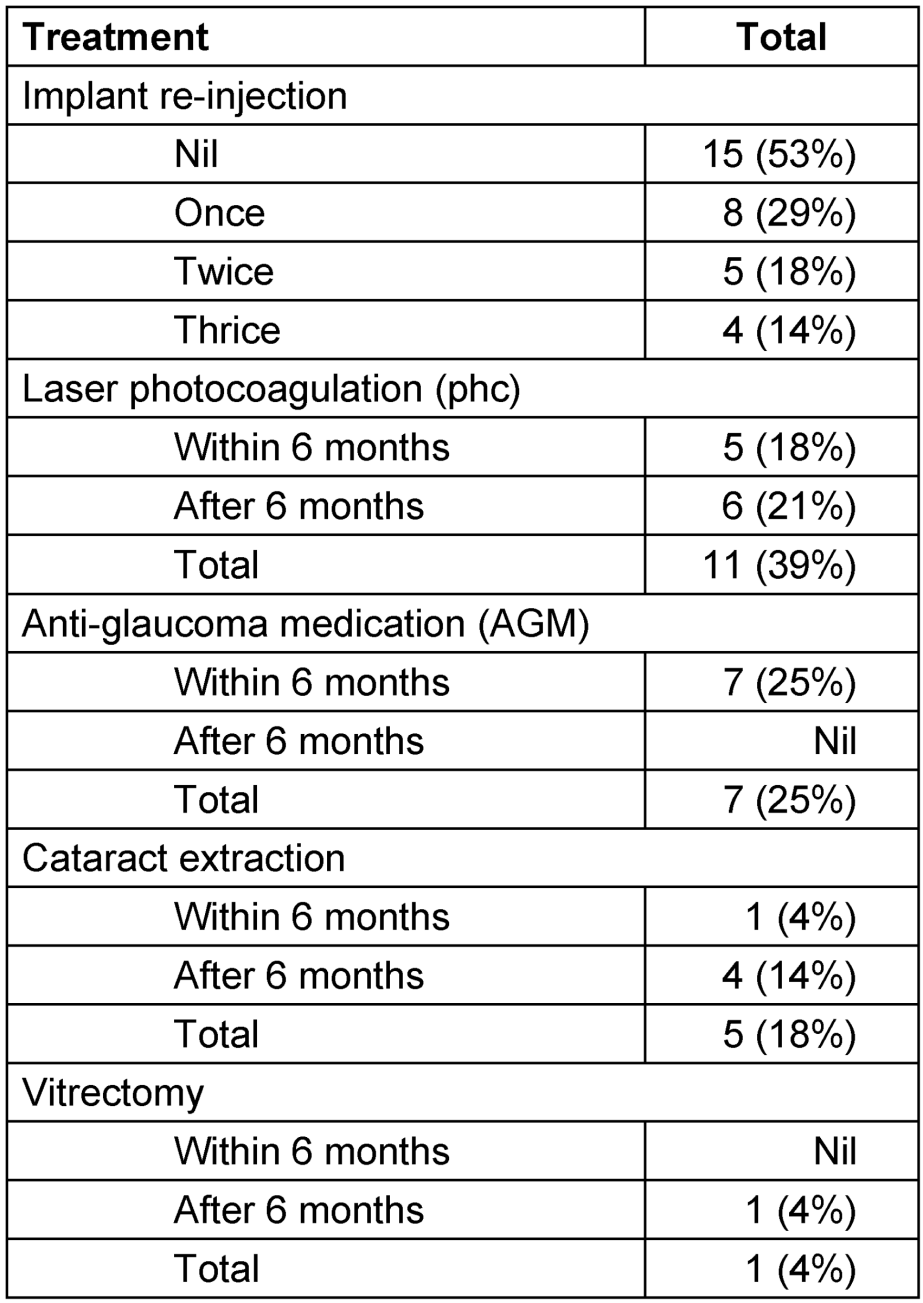
Intravitreal dexamethasone implant for refractory diabetic macular edema in 28 eyes: additional treatments during study period

**Table 3:**
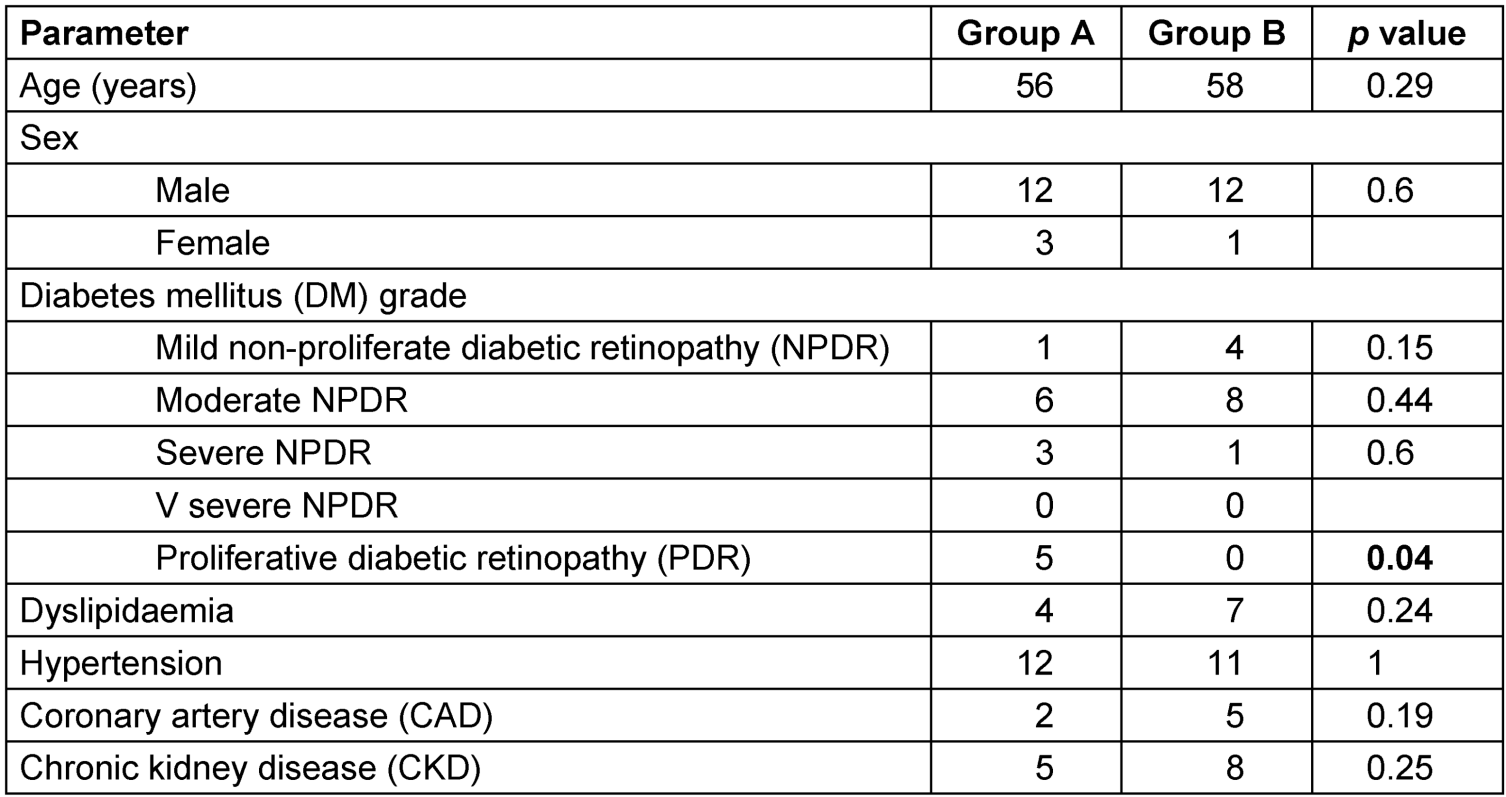
Intravitreal dexamethasone implant for refractory diabetic macular edema: comparison of systemic parameters between the two groups at presentation
